# The Impact of Paradoxical Leadership on Employee Voice Behavior: A Moderated Mediation Model

**DOI:** 10.3389/fpsyg.2020.537756

**Published:** 2020-09-24

**Authors:** Xiyuan Li, Ying Xue, Hao Liang, Dong Yan

**Affiliations:** Economics and Management School of Wuhan University, Wuhan University, Wuhan, China

**Keywords:** paradoxical leadership, voice behavior, self-efficacy, psychological safety, regulatory focus

## Abstract

Paradoxical leadership is associated with positive behavioral outcomes. However, the link between paradoxical leadership and voice behavior is not comprehensively studied in extant literature. This paper builds a theoretical model to reveal how paradoxical leadership facilitates promotive and prohibitive voice behavior of employees, drawing upon social cognitive theory and regulatory focus theory. We proposed a moderated mediation model that employees’ voice behavior is related to paradoxical leadership through self-efficacy and psychological safety. With data from 268 leader – employee pairs of questionnaires, this study conducted a structural equation model to test the conceptual framework. The results show that (a) leader’s paradoxical leadership related to employee’s promotive and prohibitive voice behaviors positively; (b) employee’s self-efficacy and psychological safety mediate the extent of effect the superior’s paradoxical leadership has on subordinate’s voice behavior; (c) the more obvious the subordinate’s promotion focus orientation, the stronger the mediating effect of self-efficacy; and (d) the more obvious the subordinate’s prevention focus orientation, the weaker the mediating effect of psychological safety. These conclusions reveal the influencing mechanism of a superior’s paradoxical leadership on a subordinate’s voice behavior. It expands paradoxical leadership-related studies, enriches studies related to the field of “leader – employee voice behavior,” and highlights the relationship between the duality of paradoxical leadership behavior on employees with different regulatory focus orientation with a new perspective.

## Introduction

Voice behavior (Van Dyne and LePine, 1998) has attracted plenty of attention in the field of organizational behavior. With the advent of artificial intelligence and the rapid growth of technology, the business environment is continually shifting. In order to ensure that organizations endure, it is necessary for superiors in an organization to take a broad and long-term view and observe the overall situation. Primarily, every employee is supposed to utilize their own wisdom, intelligence, and perform appropriate actions to provide clear advice because empirical studies have found that voice behavior is critical for sustainable development and organizational innovation (Morrison, 2014). However, voice behavior may be understood as challenging the *status quo* and as something that may have negative consequences (Son, 2019). Thus, many employees may be cautious about making a voice behavior, be reticent, or are likely to reflect before offering suggestions. As a result, many scholars focus on researching how to make employees actively engage in voice behaviors (Weiss and Morrison, 2018).

Leadership has been recognized as a key factor in the analysis of voice behavior-related risks and decision-making (Giessner and Schubert, 2007; Liu et al., 2013). Numerous scholars have studied the relationship between leadership and employee voice behaviors (LePine and Van Dyne, 2001; Detert and Burris, 2007). Leadership factors can be divided into positive and negative. Positive factors include ethical, authentic paternalistic, transformational, inclusive, servant leadership (Detert and Burris, 2007; Hsiung, 2012; Zhang et al., 2015; Chen and Hou, 2016; Yan and Xiao, 2016; Weiss et al., 2017) that can stimulate employee voice behavior. The negative factors, such as abusive leadership (Xu et al., 2015), can be detrimental. However, these studies focused on specific characteristics of leaders but did not explain how they considered managing an evolving and complicated environment. There are no relevant studies in this field that have examined the relationship between paradoxical leadership and employee voice behavior (see Table 1). Many authors had examined the influence of psychological safety on the relationship between leaders and employee voice behaviors (Liang et al., 2012, see Table 1). However, the courage required to engage in voice behavior was determined by employees’ trust in the organizational environment and their confidence in voice effectiveness, specifically the employee’s self-efficacy (Bandura, 1985). Lastly, there is little agreement among academics about how employees, with different regulatory focus tendencies, react to paradoxical leadership behaviors. Therefore, a gap in the literature exists concerning the duality of paradoxical leadership behavior with employees with different regulatory focus orientations.

**TABLE 1:** Empirical research on leadership and voice behavior.

Study	Sample	Antecedent Variables	Mediates/Moderates	Control Variables
∙ Walumbwa and Schaubroeck (2009); Walumbwa et al. (2012), Chen and Hou (2016)	∙ 291 employees on 58 teams of a government R&D institution in Taiwan ∙ 894 employees and their 222 immediate supervisors in a large financial institution in the southwestern United States ∙ 316 nurses working at a large medical center in the United States	Ethical Leadership	∙ / ∙ Psychological Safety ∙ Group Conscientiousness	∙ Gender, tenure and education ∙ Leader’s span, idealized influence leadership ∙ Group size, idealized influence leadership
∙ Wong et al. (2010); Hsiung (2012), Liang (2017)	∙ 70 work groups with 404 salespersons of chain stores in Taiwan ∙ 457 subordinates and 90 supervisors from 90 functional work groups in a large public sector organization in Taiwan ∙ 280 registered nurses working in acute care teaching and community hospitals in Ontario	Authentic Leadership	∙ Leader–Member Exchange, Positive Mood ∙ Organization-Based Self-Esteem ∙ Trust in Manager, Work Engagement	∙ Gender, age, education, marital status, organizational tenure, group size ∙ Supervisor sex, education level, age, work group tenure, and subordinate–supervisor dyad tenure, group size, psychological safety ∙ Gender, employment status, type of hospital, education, specialty area
∙ Ning et al. (2012); Chan (2014), Zhang et al. (2015)	∙ 402 leader–subordinate paired data from four companies located in Beijing in Mainland China ∙ 202 leader–follower dyads of a manufacturing factory in the People’s Republic of China ∙ 31 supervisors and 245 beauticians of a large beauty chain in Shenzhen and Zhuhai City in China	Paternalistic Leadership	∙ Leader–Member Exchange, Status Judgment ∙ Information Sharing ∙ Collectivism, Team Collectivism	∙ Subordinates’ age, gender, educational level ∙ Sex, age, education levels, organizational tenure, dyadic leader–follower tenure ∙ Age, gender, education, and marriage
∙ Detert and Burris (2007); Liu et al. (2010), Liu and Liao (2013); Duan et al. (2016)	∙ 3153 crew members in 105 restaurants ∙ 324 part-time MBA students from two universities in China ∙ 394 matched pairs of subordinates and their direct leader in Southeastern China ∙ 923 leader and follower dyads in China and Australia	Transformational Leadership	∙ Perceived Psychological Safety ∙ Social Identification, Personal Identification ∙ Leader Voice Expectation, Voice Role Perception ∙ Power Distance, Structure Distance	∙ Tenure, ethnicity, job type, gender, hours worked per week, job shift, proactive personality, employee attitudes, having ideas ∙ / ∙ Gender, age, and working tenure with leader, psychological safety, felt responsibility to change ∙ Time with leader, ethnicity
∙ Carmeli et al. (2010); Weiss et al. (2017), Qi and Liu (2017)	∙ 126 participants participated in the hospital’s 1 day simulation training sessions ∙ 150 employees who were employed in the R&D units of 8 knowledge-intensive organizations that develop advanced technological products ∙ 329 employees from 105 teams of enterprises from six major cities in China	Inclusive Leadership	Leader Language ∙ Psychological Safety ∙ Caring Ethical Climate	Team size, total number of leaders’ utterances ∙ Tenure, age ∙ Gender, age, education, tenure
∙ Walumbwa et al. (2010); Hunter et al. (2013), Yan and Xiao (2016)	∙ 473 matched supervisor subordinate dyads questionnaires ∙ 815 full-time employees from seven multinational companies operating in Kenya ∙ 337 executives from 385 stores in the US	Servant Leadership	∙ Psychological Safety ∙ Procedural Justice Climate, Employee Self-efficacy, Employee Commitment to the supervisor, Service Climate ∙ Service Climate	∙ Gender, age, politics status, tenure, highest educational ∙ / ∙ Number of employees per store
∙ Burris et al. (2008); Xu et al. (2015)	∙152 employees working in the service industry in Macau, People’s Republic of China ∙ 499 restaurant managers in United States	Abusive Leadership	∙ Emotional Exhaustion ∙ Psychological Attachment	∙ Subordinates’ gender, organizational tenure, psychological safety, affective commitment ∙ Organization tenure, position tenure, hours per week, distributive justice, alternative employment, have ideas, psychological safety, futility

This research aims at assessing the relationship between paradoxical leadership and employee voice behavior, examining the mediated effects of psychological safety and self-efficacy and the moderated effects of promotion and prevention focuses. It was propounded that the integrated social cognitive theory (Bandura, 1993) and regulatory fit theory (Higgins, 2000) explain how paradoxical leadership affects employee voice behavior. According to social cognitive theory, people observe and study an environment, which affects their behavior by influencing their objective, self-efficacy, and other factors. Paradoxical leadership can consider the needs of all sides in an organization and motivate employees to solve challenging difficulties. By studying paradoxical leadership behaviors, employees could make the correct and timely decision, increasing their self-efficacy and, hence, provide advice. Therefore, in addition to psychological safety, this study selected self-efficacy as a mediating variable of the model to explain why paradoxical leadership is related to employee voice behavior. Individuals with a dissimilar regulatory focus had their own regulating system, which included promotion and prevention focuses (Higgins, 1997). It is argued that the employee regulatory focus moderates the relationship between paradoxical leadership and employee voice behavior.

In order to test the proposed model, a pairing study was conducted, and it was found that paradoxical leadership positively relates to employees’ promotive and prohibitive voice behavior, and employee’s self-efficacy and psychological safety has a mediating role in such relationship. It was found that the more obvious the employee’s promotion focus, the stronger the mediating effect of self-efficacy, and the more obvious the employee’s prevention focus, the weaker the mediating effect of psychological safety. This study makes important contributions to the literature on employee voice, enriching the study related to the field of “leader – employee voice behavior.” Additionally, this study constructed the influencing mechanism of paradoxical leadership on employee voice behavior and expanded paradoxical leadership-related studies. Lastly, the research provided the study of the influence of the duality of paradoxical leadership behavior on employees with different regulatory focus orientation with a new perspective, which is of great significance for both theoretical development and practical management.

## Literature Review and Hypotheses

### Paradoxical Leadership and Employee Voice Behavior

Zhang et al. (2014) conceptualized paradoxical leadership behavior based on the unity of opposites in the philosophy of Yin-Yang, which has attracted the attention of many scholars and practitioners. According to them, paradoxical leadership suggests that the behavior of management, while appearing paradoxical, is internally connected and could manage the needs of organizational development and the individual needs of employees. Paradoxical leadership primarily has five dimensions:

(1) Combining self-centeredness with other-centeredness,
(2) Maintaining both distance and closeness,
(3) Treating subordinates uniformly while allowing individualization,
(4) Enforcing work requirements while allowing flexibility, and
(5) Maintaining decision control while allowing autonomy (Zhang et al., 2014, p. 541).

Voice behavior is defined as “promotive behavior that emphasizes the expression of constructive challenge intended to improve rather than merely criticize,” and as “making innovative suggestions for change and recommending modifications to standard procedures even when others disagree” (Van Dyne and LePine, 1998, p. 109). It is an active behavior that reflects the employees’ orientation to participate in discussing organizational reform and to propose constructive opinions. However, it has occasionally been necessary for the employees to identify difficulties and challenge others to improve organizational, operational efficiency, as employee voice behavior entails certain social risks (Milliken et al., 2003; Detert and Burris, 2007; Detert and Trevino, 2010; Grant, 2013). According to Liang et al. (2012), voice behavior can be categorized as promotive and prohibitive. Promotive voice behavior refers to the ability of employees to freely express their innovative ideas and suggestions to increase operational efficiency (Liang et al., 2012), while prohibitive voice behavior occurs when employees present their concerns and misgivings about problems associated with the *status quo* of their organization and propose related suggestions (Liang et al., 2012).

Leadership behavior is considered a key factor for employees in their analysis of the risks caused by voice behavior (Detert and Burris, 2007). Employees adopt different voice behavior strategies for different leadership styles (Mowbray et al., 2015). This study asserts that paradoxical leadership is related to employee voice behavior positively. Based on social cognitive theory, the organizational environment influences employees’ cognition. The management style, personality traits, and behavioral pattern of superiors affect employees’ cognition and influence their behaviors further (Detert and Burris, 2007). Additionally, paradoxical leadership makes the employees feel that their work is respected and enthuses them to provide suggestions (Liang et al., 2012). Paradoxical leaders maintain a sound relationship with employees motivating them (Mowbray et al., 2015), allowing them to be individuals, helping arouse their passion for work and job engagement, and promoting them to offer advice and suggestions for organizational development (Wang and Jiang, 2015; Lapointe and Vandenberghe, 2018). Paradoxical leadership allows flexibility at work, which can facilitate employee voice behavior (Zhang et al., 2014), stimulate employees’ sense of responsibility, and increase their intrinsic motivation to provide advice (Zhang and Bartol, 2010). Hence, the five dimensions of paradoxical leadership influence employee voice behavior positively.

According to social cognitive theory, the perceived attitudes of employees influence their behaviors. If employees believe that managers are concerned about their benefits and interests, then they would conduct extra-role performance behaviors, such as voice behavior (Walumbwa et al., 2010). Paradoxical leadership balances the conflicts between organizational and employee development and allows employees to feel supported, autonomous, and responsible. All these factors contributed to employee voice behavior.

Lastly, paradoxical leadership does not balance the contradictions in organizational management without flexibility, openness, and a willingness to learn (Zhang et al., 2015). In the process of having a good relationship with employees, paradoxical leaders also tend to be role models for employees. Employees can learn how to balance the contradictory relationship between benefits and risks of voice behavior and promote them to express their opinions at an appropriate time.

Based on the above discussions, this study proposes the following hypotheses:

Hypothesis 1a: Superior’s paradoxical leadership positively related to employee promotive voice behavior.

*Hypothesis 1b: Superior’s paradoxical leadership positively related to employee prohibitive voice behavior*.

### Mediating Effects of Self-Efficacy

Self-efficacy is defined as “people’s beliefs of their capabilities to organize and execute the courses of action required to manage prospective situations” (Bandura, 1995, p. 2). It is believed that the stronger the employee’s self-efficacy, the more likely they are to take action and be confident in their decisions (Okpozo et al., 2017; Hans and Gupta, 2018). If an employee’s self-efficacy was high, they would make greater efforts and insist action is taken in the face of possible negative results and social risks (Parker et al., 2006). Therefore, a correlation exists between higher self-efficacy and the likelihood to provide advice.

According to Wang et al. (2015), leadership is one of the most crucial external factors that influence and promotes employee self-efficacy. Specifically, paradoxical leadership balances the relationship between self-centeredness and other-centeredness by making employees feel respected, which strengthened their self-belief in their own abilities. This contributes to the formation of employee self-efficacy (Owens and Hekman, 2012). Besides, paradoxical leaders that maintain an appropriate distance from employees establish a good relationship with them, facilitating greater leader – employee interaction. A higher number of interactions imply that more information was shared, hence further increasing employee self-efficacy (Gardner et al., 2005). Furthermore, paradoxical leadership provides employees space to be more individual with increased support, allowing for flexible working, while providing a fairer environment for both leaders and employees. These factors increase employee self-efficacy. Additionally, paradoxical leadership ensures that employers maintain control over decision-making while moderately empowering employees. When employees perceive greater autonomy, their intrinsic motivation for work strengthened, enhancing their self-efficacy (Ou et al., 2014). Lastly, leaders’ own behavior sets an example for employees (Walumbwa et al., 2011) and balances uncertain management environments, contradictions, and tensions, addressing numerous challenges and increasing employee self-efficacy.

Leadership behaviors influence employee self-efficacy and play a vital role in the relationship between the leader and voice behavior (Chen and Hou, 2016). Previous studies have proven that self-efficacy mediates the relations between ethical and paternalistic leadership types and voice behavior (Wang et al., 2015). Given these discussions, the following hypotheses are acknowledged:

Hypothesis 2a: Employee’s self-efficacy mediates the relationship between paradoxical leadership and promotive voice behavior.

*Hypothesis 2b: Employee’s self-efficacy mediates the relationship between paradoxical leadership and prohibitive voice behavior*.

### Mediating Effects of Psychological Safety

According to previous literature, employees might weigh or consider the advantages and disadvantages of voice behavior because of its potential negative effects. According to Kahn (1990), individuals who had high psychological safety were assured of their status and hence provided advice about possible misunderstandings, negative impressions, and the positive effects caused by the external environment, without concern. With psychological safety, employees’ misgiving about the possible negative effects of voice behavior was lessened, and they felt assured about expressing their own opinion. Furthermore, Liang et al. (2012) also indicated that the higher the psychological safety based on organizational cognition was, the more likely employees were to work on voice behaviors.

Based on the social cognitive theory, employees’ motives, attitudes, and behaviors are influenced by the environment. When making the decision to provide advice, employees carefully evaluate whether the surrounding environment is favorable for voice behavior, as employees were more likely to provide advice in a supportive and superior interpersonal environment (Carmeli et al., 2009). When leaders were uninterested in employee voice behavior, employees sensed inconsistencies with their leader’s expectation and hence generated psychological insecurity. But when leaders presented more instructive, supportive, and open characteristics, had a sound relationship with employees, and took their individual needs into account, they gained the trust of their employees and increased their psychological safety (Liang et al., 2012).

Detert and Burris (2007) realized that the psychological safety of employees mediated and explained the relations between the openness of the leader and the voice behavior of the employee. When employees felt their opinions were supported by their superiors, their psychological safety increased, encouraging them to express their views (Nembhard and Edmondson, 2006). When leaders meet both the needs of the organization and employees, they respect the employees’ individuality, maintain their self-esteem and confidence, and capitalize on their advantages and abilities. These characteristics directly facilitate harmonious interpersonal relationships with employees, build a supportive and open environment for leaders and employees, improve employees’ psychological safety, and encourage them to provide advice to paradoxical leaders (Mowbray et al., 2015).

Several studies have examined the link between leadership and psychological safety. For instance, Walumbwa and Schaubroeck (2009) stated that psychological safety involves perceiving and experiencing a high degree of interpersonal trust. Further, psychological safety takes a central stage in the context of work environment wherein employees develop mutual trust as well as comfort level in working with each other, both of which indirectly affect their voice behavior. Yang et al. (2019) also mentioned that psychological safety is a key differentiating factor that encourages creativity at workplace and provides additional insights about the paradoxical leadership behavior. Authors have also suggested that by undermining hierarchical barriers, leaders encourage a climate of psychological safety and trust wherein employees feel more confident and safer not only while sharing suggestions but also while raising any work-related issues (Edmondson, 2003; Nembhard and Edmondson, 2006; Hsiung, 2012). In the abovementioned studies, the authors have considered psychological safety as a unidimensional construct and mediating variable. Hence, this study illustrates the following hypotheses:

*Hypothesis 3a: Employees’ psychological safety mediates the relationship between paradoxical leadership and promotive voice behavior*.

*Hypothesis 3b: Employees’ psychological safety mediates the relationship between paradoxical leadership and prohibitive voice behavior*.

### Moderating Effects of Regulatory Focus

According to a study performed by Higgins (1997), there are two self-regulating systems – promotion focus and prevention focus. Individuals with unique self-concept and different regulatory focus orientation have a different emotional experience, cognitive style, and action. Individuals with greater promotion focus pay more attention to vision, expectation, and gains; are more sensitive to rewards; and reveal the pursuit of their “ideal self.” People who are with a prevention focus concentrated on duty, responsibility, and losses; are more sensitive to punishment; and reveal their pursuit of the rules and the realization of their “moral self.”

According to regulatory fit theory, if external influences matched the characteristics of individual regulatory focus orientation, the external influence was strengthened. Specifically, if employees reasoned that the leadership style was consistent with their regulatory focus orientation, it may stimulate the generation of a fitted regulator, influence the employees’ cognition, and strengthen their behavioral motive. If employees believe that leadership style is inconsistent with their regulatory focus orientation, it would be challenging to generate regulatory fit and the employees’ cognition, and managers would fail to influence their behavioral motive (Henker et al., 2015).

In relation to the effect of paradoxical leadership on employee behavior, superior’s paradoxical leadership centered on balancing the contradictions and tensions between organizational and employee needs, making employees feel the different characteristics of the leaders. Specifically, paradoxical leadership is employee-centered and provides employees with moderate flexibility, empowerment, and individuation. Such behaviors helped employees pursue their “ideal self.” This self-concept prompts employees to implement behavioral strategy, which corresponds with employee promotion focus orientation, enhances their self-efficacy, and increases their confidence to perform voice behavior. Conversely, paradoxical leadership ensures employees meet their job requirements, controls decision-making, and ensures that all employees are treated fairly. These behavioral characteristics are closely aligned with employees’ “moral selves.” This self-concept prompts employees to perform “avoidant” behavioral strategies, which accords with the prevention focus orientation of employees. For employees with an intensive prevention focus, their behavioral strategy remained more cautious and conservative and was less affected by the external environment (Lee et al., 2000). Paradoxical leadership contradicted the clear rules, responsibilities, and duties that they observed and was more likely to be seen as a violation of the rules, resulting in lower self-efficacy, psychological safety, and a decrease in their voice behaviors.

Hence, the following hypotheses are proposed based on previous literature:

Hypothesis 4a: Employee’s promotion focus positively moderates the mediating effect of employee’s self-efficacy in the relationship between paradoxical leadership and voice behavior; the higher the promotion focus, the stronger the mediating effect of self-efficacy.

*Hypothesis 4b: Employee’s prevention focus negatively moderates the mediating effect of employee’s psychological safety in the relationship between paradoxical leadership voice behaviors; the higher the prevention focus, the weaker the mediating effect of psychological safety*.

**FIGURE 1:**
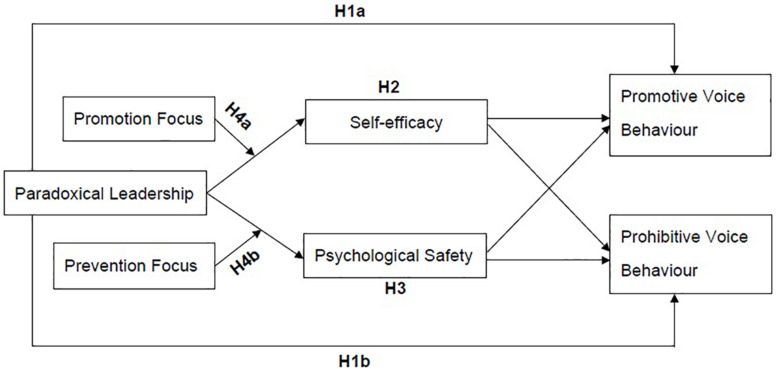
Illustrates the research model and all hypotheses of this research.

Figure 1 illustrates the research model and all hypotheses of this research.

## Materials and Methods

### Participants and Procedure

A total of 360 leader – employee pairs of questionnaires were distributed in several cities in China, including Beijing, Hangzhou, Wuhan, Anhui, Zhengzhou, Shenzhen, and Guangzhou, to white-collar workers from a variety of industries such as finance, communication, manufacturing, education, and real estate.

After removing the respondents with regular responses, missing items, and ineligible details, we finally obtained 268 valid response pairs of questionnaires that were used for analysis. These leaders are presently in work relationship with these employees or have had worked in past. Due to this, the data collected from these leaders and employees helped in reaching more insightful results based on their behavioral experiences. In the sample of leaders, 63.3% were males, and 36.7% were females. They represented five age groups, namely, 18–25 years (4.4%), 26–30 years (30.0%), 31–40 years (46.7%), 41–50 years (12.2%), and over 50 years (6.7%). Among the employees, 51.1% were males and 48.9 were females. They represented five age groups, namely, 18–25 years (31.0%), 26–30 years (42.5%), 31–40 years (20.1%), 41–50 years (6.0%), and over 50 years (0.4%).

### Ethics Statement

Prior to commencing the data collection, ethical approval was applied and was approved by the Research Ethics Committee of the University. According to our research design, the study did not violate any legal regulations or common ethical guidelines. In order to ensure that this study has followed ethical principles, the research purpose of the study is introduced, and consent was obtained before completing the hard copy questionnaires. Additionally, we emphasized that all the participants could reject any questions or withdraw from the study at any time. Lastly, their anonymity and confidentiality were assured.

### Measures

#### Paradoxical Leadership

This study utilized a paradoxical leadership scale designed by Zhang et al. (2015). This scale consisted of 22 items and five dimensions, and its Cronbach’s α was 0.897. As a general research practice, a value of 0.70 or higher for Cronbach’s alpha qualifies a research instrument for self-consistence and acceptance. Employee participants were asked to report their leader’s paradoxical leadership on a five-point Likert scale. An example item was: “Uses a fair approach to treat all employees uniformly, but also to treat them as individuals.” Response options ranged from (1) strongly disagree to (5) strongly agree.

#### Self-Efficacy

The general self-efficacy scale designed by Schwarzer et al. (1997) was adopted in this study. This scale contained 10 items, and its Cronbach’s α was 0.946. Employee participants self-reported their self-efficacy on a five-point Likert scale. An example item was: “I will be able to achieve most of the goals that I have set for myself.” Response options ranged from (1) strongly disagree to (5) strongly agree.

#### Psychological Safety

A five-item scale designed by Liang et al. (2012) was used to measure psychological safety. Employee participants were asked to reported their psychological safety on a five-point Likert scale. Its Cronbach’s α was 0.889. An example item was: “In my work unit, I can express my true feelings regarding my job.” Response options ranged from (1) strongly disagree to (5) strongly agree.

#### Promotive Voice Behavior

This study adopted the five-item scale designed by Liang et al. (2012) to measure promotive voice behavior, and its Cronbach’s α was 0.925. Leader participants rated their employee’s promotive voice behavior on a five-point Likert scale. An example item was: “Proactively develop and make suggestions for Nos that may influence the unit.” Response options ranged from (1) strongly disagree to (5) strongly agree.

#### Prohibitive Voice Behavior

The five-item scale designed by Liang et al. (2012) was utilized in this study, and its Cronbach’s α was 0.859. Leader participants rated their employee’s prohibitive voice behavior on a five-point Likert scale. An example item was: “Advise other colleagues against undesirable behaviors that would hamper job performance.” Response options ranged from (1) strongly disagree to (5) strongly agree.

#### Regulatory Focus

This study uses the regulatory focus scale designed by Zhou et al. (2012). This scale consists of seven items and two dimensions. Promotion focus has four items. An example item is “In general, I am focused on achieving positive outcomes in my life.” The Cronbach’s α was 0.875. Prevention focus included three items, for example, “I often worry that I will fail to accomplish my work goals.” The Cronbach’s α was 0.863. Employee participants were asked to rate their regulatory focus on a five-point Likert scale (“1” = “strongly disagree” and “5” = “strongly agree”).

#### Control Variables

This study collects demographics of leaders and employees, including gender, working years, and age, to use as control variables and recoded every variable. In terms of gender variable, a male was coded as “1” and female was coded as “2.” For work experience, less than 3 years’ experience was coded as “1,” 4–5 years was coded as “2,” 6–10 years was coded as “3,” 11–20 years was coded as “4,” and over 20 years was coded as “5.” In relation to age, those aged 18–25 were coded as “1,” aged 26–30 were coded as “2,” aged 31–40 were coded as “3,” aged 41–50 were coded as “4,” and those over 50 were coded as “5.”

## Data Analysis and Results

To establish the cause and relationship among the variables conceptualized in this study, the following procedures were adopted: Firstly, Amos 20.0 software was used to perform the confirmatory factor analysis to investigate the discriminant validity of the variables. Then, this study utilized Herman’s single-factor test to conduct common method biases analysis. Following that, SPSS 22.0 was adopted to conduct descriptive statistical analysis. Lastly, the study utilized Mplus 7.0 to verify hypotheses.

### Confirmatory Factor Analysis

In order to investigate the discriminant validity of the variables in the theoretical model, Amos 20.0 software was utilized to perform the confirmatory factor analysis. A factor packing method was used to build models with different factors, and they were compared in terms of goodness of fit. The results in Table 2 indicate that the seven-factor model was the most effective.

**TABLE 2:** Results of confirmatory factor analysis.

Model		*CMIN/df*	*RMSEA*	*GFI*	*CFI*	*TLI*
Seven-factor	*PL,PE,PS,V1,V2,F1,F2*	1.489	0.043	0.911	0.976	0.971
Six-factor	*PL,PE* + *PS,V1,V2,F1,F2*	3.153	0.09	0.824	0.893	0.872
Five-factor	*PL,PE* + *PS,V1* + *V2,F1,F2*	3.402	0.095	0.806	0.878	0.857
Four-factor	*PL,PE* + *PS,V1* + *V2,F1* + *F2*	5.402	0.128	0.731	0.772	0.739
Three-factor	*PL* + *PE* + *PS,V1* + *V2,F1* + *F2*	6.047	0.137	0.709	0.735	0.7
Two-factor	*PL* + *PE* + *PS,V1* + *V2* + *F1* + *F2*	9.025	0.173	0.597	0.574	0.523
Single-factor	*PL* + *PE* + *PS* + *V1* + *V2* + *F1* + *F2*	12.295	0.206	0.509	0.398	0.329

*PL, Paradoxical Leadership; PE, Self-efficacy; PS, Psychological Safety; V1, Promotive Voice Behavior; V2, Prohibitive Voice Behavior; F1, Promotion Focus; F2, Prevention Focus. “ + ” refers to “combine two factors into a single factor.”*

### Common Method Biases Analysis

Two measures were utilized to remit the effect of common method bias in this study. On the one hand, experienced academicians were invited to check all items on our questionnaire and revise explanations. Respondents were allowed to, anonymously, comment when completing the questionnaires. On the other hand, Herman’s single-factor test was conducted on the survey data, and a factor analysis was performed on all questionnaire items with SPSS v22.0. The initial characteristic value of 11 factors was higher than 1, the explained total variation was 70.69%, and the first factor was 21.19%, which was lower than totality by 50%, so common method biases were acceptable.

### Descriptive Statistical Analysis

SPSS. v22.0 software was utilized for the descriptive analysis of the variables in the study, and the results are presented in Table 3. The results revealed that there were significant positive correlations between paradoxical leadership and (a) employee promotive voice behavior (*r* = 0.158, *P* < 0.01), (b) employee prohibitive voice behavior (*r* = 0.158, *P* < 0.01), (c) employee self-efficacy (*r* = 0.181, *P* < 0.01), and (d) employee psychological safety (*r* = 0.169, *P* < 0.01). These were consistent with the hypotheses proposed in this study.

**TABLE 3:** Results of descriptive statistical analysis.

Variable	M	*SD*	*PL*	*PE*	*PS*	*V1*	*V2*	*F1*	*F2*
Paradoxical leadership	3.888	0.475	1						
Self-efficacy	3.628	0.735	0.181**	1					
Psychological safety	3.590	0.828	0.169**	0.258**	1				
Promotive voice behavior	3.796	0.776	0.206**	0.320**	0.250**	1			
Prohibitive voice behavior	3.713	0.699	0.158**	0.346**	0.287**	0.752**	1		
Promotion focus	3.794	0.811	0.139*	0.456**	0.115	0.120*	0.192**	1	
Prevention focus	3.039	0.949	0.176**	0.117	0.075	0.145*	0.116	0.013	1

PL, Paradoxical Leadership; PE, Self-efficacy; PS, Psychological Safety; V1, Promotive Voice Behavior; V2, Prohibitive Voice Behavior; F1, Promotion Focus; F2, Prevention Focus. *p < 0.05. **p < 0.01.

### Hypothesis Testing

**FIGURE 2:**
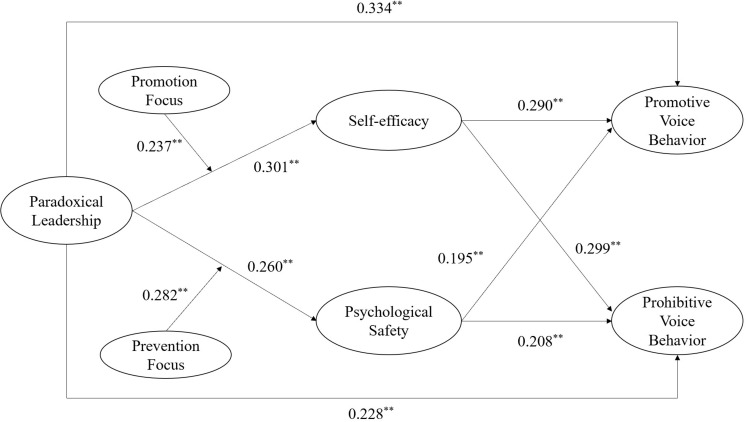
Path Coefficients of the Hypothesis Model.

Mplus 7.0 was utilized to construct a structural equation model, and the SEM fitness indexes of χ^2^/df, RMSAEA, SRMR, CFI, and TLI (χ^2^/df = 1.949, RMSAEA = 0.060, SRMR = 0.033, CFI = 0.969, and TLI = 0.919) all met the recommended values, which indicated that the model constructed in this study was reasonable and reliable, which could be analyzed in the next step. The structural model with path coefficients is presented in Figure 2.

It can be seen in Figure 2 that the path coefficient of paradoxical leadership on promotive voice behavior was positive and significant (β = 0.334, *p* < 0.01), supporting hypothesis 1a. The path coefficient of paradoxical leadership on prohibitive voice behavior was positive and significant (β = 0.228, *p* < 0.01), which supported hypothesis 1b.

Table 4 illustrated the results of mediating effects, which is based on the bootstrapping method. As shown in Table 4, the 95% confidence interval for bias correction of the direct effect of self-efficacy on paradoxical leadership and promotive voice behavior was [0.053, 0.242] (which did not include 0). This result indicated that the indirect effect of paradoxical leadership on promotive voice behavior was significant, which means that self-efficacy mediated the relationship between paradoxical leadership and promotive voice behavior, supporting hypothesis 2a. The 95% confidence interval for bias correction of the direct effect of self-efficacy on paradoxical leadership and prohibitive voice behavior was [0.081, 0.239] (which did not include 0). This result indicated that the indirect effect of paradoxical leadership on prohibitive voice behavior was significant, which means that self-efficacy mediated the relationship between paradoxical leadership and prohibitive voice behavior, supporting hypothesis 2b.

**TABLE 4:** Results of mediating effect.

Effect	Estimate	SE	Est./SE	BC 95% CI	
				Lower	Upper
**PL-PV1**					
Total effect	0.210	0.089	2.363	0.036	0.383
Direct effect	–0.012	0.095	–0.128	–0.203	0.175
**Indirect effect**					
TOTAL	0.222	0.052	4.300	0.109	0.323
*PL-PS-PV1*	0.084	0.032	2.659	0.134	0.347
*PL-PE-PV1*	0.138	0.048	2.905	0.053	0.242
**PL-PV2**					
Total effect	0.149	0.067	2.236	0.025	0.294
Direct effect	–0.098	0.070	–1.395	–0.226	0.043
**Indirect effect**					
TOTAL	0.247	0.046	5.333	0.171	0.348
*PL-PS-PV2*	0.098	0.029	3.322	0.045	0.162
*PL-PE-PV2*	0.149	0.041	3.657	0.081	0.239

*PL, Paradoxical Leadership; PE, Self-efficacy; PS, Psychological Safety; V1, Promotive Voice Behavior; V2, Prohibitive Voice Behavior.*

The 95% confidence interval for bias correction of the direct effect of psychological safety on paradoxical leadership and promotive voice behavior was [0.134, 0.347] (which did not include 0). This result indicated that the indirect effect of paradoxical leadership on prohibitive voice behavior was significant, which means that psychological safety mediated the relationship between paradoxical leadership and promotive voice behavior, supporting hypothesis 3a. The 95% confidence interval for bias correction of the direct effect of psychological safety on paradoxical leadership and prohibitive voice behavior was [0.045 0.162] (which did not include 0). This result indicated that the indirect effect of paradoxical leadership on prohibitive voice behavior was significant, which means that psychological safety mediated the relationship between paradoxical leadership and promotive voice behavior, supporting hypothesis 3b.

On the basis of the mediating effects, promotion focus and prevention focus were taken as moderated variables, and the SEM was reconstructed. The fitness indexes of χ^2^/df, RMSAEA, SRMR, CFI, and TLI (χ^2^/df = 1.944, RMSAEA = 0.059, SRMR = 0.029, CFI = 0.965, and TLI = 0.896) all met the recommended values, which indicated that the model constructed in this study was reasonable and reliable, which could be analyzed in the following step.

In Table 5, when high promotion focus orientation was obvious, the mediating effect of employee self-efficacy in the relationship between paradoxical leadership and employee promotive voice behavior (Effect = 0.148, Boot 95% CI = [0.077, 0.372]) and employee prohibitive voice behavior was significant (Effect = 0.179, Boot 95% CI = [0.084, 0.287]). When low promotion focus orientation was obvious, the mediating effect of employee self-efficacy in the relationship between paradoxical leadership and employee promotive voice behavior was non-significant (Effect = 0.084, Boot 95% CI = [−0.075, 0.260]). Additionally, its mediating effect in the relationship between paradoxical leadership and employee prohibitive voice behavior was non-significant (Effect = 0.083, Boot 95% CI = [−0.038, 0.240]). This indicated that the focus of the promotion positively regulated the mediating effect of self-efficacy in the relationship between paradoxical leadership and employee voice behavior.

**TABLE 5:** Results of moderated mediation analysis.

Mediators	Dependent variables	Moderators	Level	Effect	Boot SE	Boot 95% CI	
						Lower	Upper
*PE*	*V1*	*F1*	M−1 *SD*	0.084	0.064	–0.075	0.260
			M	0.112	0.052	0.036	0.294
			M + 1 *SD*	0.148	0.058	0.077	0.372
	*V2*	*F1*	M−1 *SD*	0.083	0.069	–0.038	0.240
			M	0.109	0.054	0.017	0.261
			M + 1 *SD*	0.179	0.062	0.084	0.287
*PS*	*V1*	*F2*	M−1 *SD*	–0.024	0.038	–0.118	0.039
			M	–0.083	0.053	–0.242	0.016
			M + 1 *SD*	–0.107	0.051	–0.296	–0.001
	*V2*	*F2*	M−1 *SD*	–0.032	0.050	–0.141	0.062
			M	–0.094	0.046	–0.248	–0.033
			M + 1 *SD*	–0.156	0.041	–0.375	–0.011

*PL, Paradoxical Leadership; PE, Self-efficacy; PS, Psychological Safety; V1, Promotive Voice Behavior; V2, Prohibitive Voice Behavior; F1, Promotion Focus; F2, Prevention Focus.*

When high prevention focus orientation was significant, the mediating effect of employee psychological safety in the relationship between paradoxical leadership and employee promotive voice behavior (Effect = −0.107, Boot 95% CI = [−0.296, −0.001]) and prohibitive voice behavior was significant (Effect = −0.156, Boot 95% CI = [−0.375, −0.011]). When low prevention focus orientation was significant, the mediating effect of employee psychological safety in the relationship between paradoxical leadership and employee promotive voice behavior (Effect = −0.024, Boot 95% CI = [−0.118, 0.039]) and employee prohibitive voice behavior was non-significant (Effect = −0.032, Boot 95% CI = [−0.141, 0.062]). This suggests that prevention focus reversely moderates the mediating effect of psychological safety in the relationship between paradoxical leadership and employee voice behavior. Therefore, hypotheses 4a and 4b were supported.

## Discussion

Based on social cognitive theory and regulatory fit theory, this study examines the influencing mechanism of paradoxical leadership on employee voice behavior, proposed by Zhang et al. (2015). It discusses how employee self-efficacy and psychological safety mediated and explained it by analyzing the regulating effect of employee regulatory focus orientation and sample data acquired from the survey. Finally, it draws conclusions corresponding to the model. Moreover, the proposed model explained and provided good fit to the data. First, it was found that both promotive voice behavior and prohibitive voice behavior can be encouraged by practicing paradoxical leadership, demonstrating positive relationship. Second, we found that there is an indirect effect of paradoxical leadership on voice behavior through self-efficacy and psychological safety. Third, when the moderating effect of regulatory focus of employees was considered into the model, it was revealed that promotion focus has a positive moderating effect on mediating effect of self-efficacy and prevention focus has an inverse moderating effect on mediating effect of psychological safety.

### Theoretical Implications

Initially, the study conducts an examination of the relationship between paradoxical leadership and employee voice behavior, expanding the previous studies pertaining to paradoxical leadership. Although prior research has examined the relationship between leadership and employee voice behaviors (LePine and Van Dyne, 2001; Detert and Burris, 2007), there is still a scarcity of studies in this area. The empirical results of this study reveal that paradoxical leadership can promote employee voice behavior. When leaders tend to engage into paradoxical leadership behavior, employees tend to engage in both promotive and prohibitive voice behaviors. This finding contributes to the development of a more comprehensive account of the relationship between paradoxical leadership and voice behavior.

The study also contributes to the existing literature about how a paradoxical style of leadership relates to employee voice behavior by highlighting the mediating effects of self-efficacy and psychological safety and moderating effects of regulatory focus in a collective manner rather than studying different factors individually and by integrating the social cognitive theory and regulatory focus theories. The empirical results suggest that paradoxical leadership balances the conflicts between organizational and employee development and allows employees to feel supported, autonomous, and responsible. This can be explained further with the help of social cognitive theory that the perceived attitudes of employees are connected to their behaviors. If employees believe that managers are concerned about their benefits and interests, then they would conduct extra-role performance behaviors, such as voice behavior (Walumbwa et al., 2010).

Despite verifying the mediating effects of psychological safety, the results of this study are strongly supportive of the mediating effects of self-efficacy as well. The majority of previous studies examining the relationship between leadership and employee voice behavior focused on the mediating effect of psychological safety. However, this study explains both employee self-efficacy and psychological safety mediated between paradoxical leadership and employee voice behavior. This conclusion reveals how paradoxical leadership is related to employee voice behavior, enriching and perfecting voice behavior-related theories. This finding supports the results conducted by Mowbray et al. (2015), which highlights that self-efficacy can mediate the relationship between voice behavior and another leadership despite ethical and paternalistic leadership (Wang et al., 2015).

Lastly, this study explored that regulatory focus moderates the mediating effect in influencing the mechanism of paradoxical leadership on employee voice behavior with the help of conclusive evidences. The findings obtained verify that individuals with a dissimilar regulatory focus behave differently, which dovetail with Higgins’s (1997) study. Besides, there is little agreement among academicians about how employees with different regulatory focus tendencies react to paradoxical leadership behaviors as argued by Lee et al. (2000) and Henker et al. (2015). Through empirical testing in this study, it was revealed that promotion focus has a positive moderating effect on mediating effect of self-efficacy and prevention focus has an inverse moderating effect on mediating effect of psychological safety. This is in contrast to the arguments presented in academic literature. For instance, as per the regulatory fit theory, the voice behavior would be strengthened in cases where leadership style was consistent with their regulatory focus orientation and vice versa (Henker et al., 2015).

In addition, it was revealed that employees who practice high regulatory focus orientation, both promotion and preventive, when their regulatory focus matches with leadership style, may stimulate the generation of a fitted regulator, employees’ cognition, and strengthen their behavioral motive. In contrast, at low levels of regulatory focus orientation, it would be challenging to generate regulatory fit with employees’ cognition, and managers would fail to influence their behavioral motive (Henker et al., 2015). Moreover, the literature also suggested that paradoxical leadership is employee-centric and helps employees to pursue their “ideal self.” These findings thus contribute to the development of a better understanding of the relationship between paradoxical leadership and voice behavior.

### Practical Implications

This study discusses the influencing mechanism of paradoxical leadership on employee voice behavior and constitutes an expansion of the studies on the topic. The conclusions drawn offer some guidance for business management practice.

Firstly, managers should improve the characteristics of paradoxical leadership behaviors. Currently, the business environment is constantly shifting and managements face uncertainty and complicated challenges. Managers in organizations are unavoidably confronted with numerous dilemmas and paradoxes. Their ability to deal with these are, thus, increasingly important to the effectiveness of leadership. While managers see these challenges from a paradoxical perspective that originate in the paradox theory, the way of thinking will expand their cognition, integrate complexities, survey the *status quo* from the perspective of long-term strategic development, and balance the contradictions and tensions with appropriate management behaviors. For example, in today’s competitive world, stress is common among the employees that originate from rising competing demands. Such demands include operating in the short term while planning for the long term, dealing with local scenarios while acting globally, and competing as well as collaborating with other firms to remain profitable yet socially and environmentally friendly. The results obtained thus suggest that leaders embracing paradoxes help employees and subordinates in making sense of such demands and voicing out their opinion to improve processes and structures.

Secondly, organizations are suggested to stress the building of the organizational atmosphere in order to improve employees’ psychological safety and self-efficacy. Managers should adjust their management style, constantly optimize their management work, attend to employees’ job performance, guide their work based on employees’ characteristics, provide employees moderate flexibility and autonomy, place emphasis on the building of team culture, provide positive feedback about employee behaviors, and execute suitable incentives. These approaches will strengthen employees’ confidence, improve the relationships between leaders and employees, improve employees’ psychological safety, and improve employees’ psychological cognition. For instance, paradoxical leadership provides employees space to be more individual with increased support, allowing for flexible working, while providing a fairer environment for both leaders and employees. These factors increase self-efficacy of employees, which in turn boosts their voice behavior.

Thirdly, the way of managing employees should lay emphasis on adjusting measures to different employees and implement proper management strategy according to personal characteristics of employees. Managers lead employees differently because of their different individual characteristics; hence, managers should pay attention to their subordinates as much as possible, implement proper management strategy, and stimulate personal potential to the maximum extent, for example, offering employees promotion to focus more on autonomy, encouraging employees to be more active in voice behavior, providing employees with prevention measures that focus more on care and personalized instructions, making job requirements clearer and more specific, relieving their doubts about risks, and promoting their psychological safety.

Lastly, paradoxical leadership is employee-centered and provides employees with moderate flexibility, empowerment, and individuation. Such behaviors helped employees pursue their “ideal self.” This self-concept prompts employees to implement behavioral strategy, which corresponds with employee promotion focus orientation, enhances their self-efficacy, and increases their confidence to perform voice behavior. However, if employees practice an intensive level of regulatory focus, their behavioral strategy remains more cautious and conservative and was less affected by the external environment (Lee et al., 2000). For example, employees with intensive prohibitive behavior may see paradoxical leaders’ competing behaviors as a clear contradiction of rules, which may undermine their self-efficacy, psychological safety, and consequently their voice behavior.

### Limitations and Recommendation

Limitations of this study should be addressed in the future. First, this study was conducted from the perspective of employee psychological cognition, self-efficacy, and psychological safety, which may be considered insufficient. In the “black box” of the relationship between paradoxical leadership and employee voice behavior, there are other interpreting mechanisms, such as the perspective of employees’ self-determination and leader–member exchange. These factors were not included in this study, and related studies may wish to consider these perspectives in the future. Secondly, due to a number of restrictions, this study used cross-section data, making it challenging to examine the validity of the theoretical model in a long-term period. Although data were collected from various sources and included both managers and employees to reduce common method biases, this study could not obtain longitudinal, multi-temporal data and did not verify the long-term effect of paradoxical leadership on employee voice behavior. Future studies may consider these aspects to extend this research.

## Data Availability Statement

The original contributions presented in the study are included in the article/supplementary material, further inquiries can be directed to the corresponding author.

## Ethics Statement

Prior to commencing the data collection, ethical approval was applied and was approved by the Research Ethics Committee of the University. According to our research design, the study did not violate any legal regulations or common ethical guidelines. In order to ensure that this study has followed the ethical principles, the research purpose of the study is introduced, and consent was obtained before completing the hard copy questionnaires. Additionally, we emphasized that all the participants could reject any questions or withdraw from the study at any time. Lastly, their anonymity and confidentiality were assured.

## Author Contributions

XL conceived the theoretical framework, organized data collection, and supervised this study. YX contributed to the research idea and data analysis, and composed the manuscript. HL and DY collected the data, analyzed the statistics, and edited the manuscript. All authors contributed to the article and approved the submitted version.

## Conflict of Interest

The authors declare that the research was conducted in the absence of any commercial or financial relationships that could be construed as a potential conflict of interest.
